# Synergistic Effects of Obesity and Hyperglycemia on Hippocampal Neurodegenerative Decline Disrupt the Neural Circuitry Regulating Motivation in Zucker Diabetic Fatty Rats

**DOI:** 10.3390/metabo16020107

**Published:** 2026-02-03

**Authors:** Martha Patricia Islas-Islas, Aleida Monserrat Coss-Orozco, Diana Moroni-González, Erick Flores-Cholula, José Everardo Avelino-Cruz, Julio Cesar Morales-Medina, Alfonso Diaz, Fabián Galindo-Ramírez, Samuel Treviño, Rubén Antonio Vázquez-Roque

**Affiliations:** 1 Laboratorio de Neuroplasticidad y Metabolismo, Instituto de Fisiología, Benemérita Universidad Autónoma de Puebla, 14 Sur 6301, Ciudad Universitaria, Puebla C.P. 72560, Mexico; martha.islas@utectulancingo.edu.mx (M.P.I.-I.); co224470443@alm.buap.mx (A.M.C.-O.); 2 Doctorado en Ciencias Biológicas, Centro de Tlaxcala de Biología de la Conducta, Universidad Autónoma de Tlaxcala, Tlaxcala C.P. 90000, Mexico; 3 Centro de Investigación en Reproducción Animal, Cinvestav-Universidad Autónoma de Tlaxcala, AP 62, Tlaxcala C.P. 90000, Mexico; diana.moroniglz@correo.buap.mx (D.M.-G.); jmoralesm@cinvestav.mx (J.C.M.-M.); 4 Laboratorio de Metabolómica y Enfermedades Crónico-Degenerativas, Instituto de Fisiología, Benemérita Universidad Autónoma de Puebla, 14 Sur 6301, Ciudad Universitaria, Puebla C.P. 72560, Mexico; eric.floresc@alumno.buap.mx; 5 Laboratorio de Cardiología Molecular, Instituto de Fisiología, Benemérita Universidad Autónoma de Puebla, 14 Sur 6301, Ciudad Universitaria, Puebla C.P. 72560, Mexico; jose.avelino@correo.buap.mx; 6 Laboratorio de Neuroquímica y Conducta, Instituto de Fisiología, Benemérita Universidad Autónoma de Puebla, 14 Sur 6301, Ciudad Universitaria, Puebla C.P. 72560, Mexico; 7 Laboratorio de Cáncer y Comunicación Celular, Instituto de Fisiología, Benemérita Universidad Autónoma de Puebla, 14 Sur 6301, Ciudad Universitaria, Puebla C.P. 72560, Mexico

**Keywords:** obesity, type 2 diabetes, motor activity, cognitive functions, hippocampal detriment

## Abstract

**Background/Objectives**: Type 2 diabetes (T2D) and obesity are chronic metabolic disorders associated with cognitive impairment and neuronal damage. The hippocampus, a region sensitive to nutrient excess, is critical for integrating sensory and metabolic signals. This study aimed to determine the early onset of cognitive and motor deficits induced by obesity and/or hyperglycemia and to characterize associated hippocampal alterations in Zucker Diabetic Fatty (ZDF) rats. **Methods:** Male ZDF rats (13 weeks old) were categorized into three groups: lean control, obese normoglycemic (ZDF-NG), and obese hyperglycemic (ZDF-HG). Assessments included zoometric parameters (weight and adiposity), biochemical assays (glucose tolerance, insulin response, and lipid profile), and behavioral tests (Open Field and Novel Object Recognition). Hippocampal health was evaluated through stereological neuronal density analysis and redox balance markers. **Results:** Both obese groups exhibited significant visceral adiposity and hyperlipidemia. The ZDF-HG group was further characterized by glucose intolerance, hepatic insulin resistance, and reduced β-cell function. Behavioral results showed that while obesity decreased motor activity, hyperglycemia significantly exacerbated the loss of both short- and long-term recognition memory. Histologically, obesity was associated with decreased neuronal density in the hippocampal DG and CA1 regions. Furthermore, hippocampal ROS was significantly elevated in the ZDF-HG group, and glutathione reductase activity was reduced in both obese phenotypes. **Conclusions:** The findings demonstrate that obesity initiates hippocampal neurodegeneration and motor decline, and that hyperglycemia severely impairs recognition memory. These results emphasize the critical interplay between metabolic dysfunction and cognitive decline, highlighting the necessity of managing both obesity and T2D to prevent early neurodegenerative changes.

## 1. Introduction

Type 2 diabetes (T2D) is a chronic metabolic disorder characterized by altered insulin secretion and variable degrees of peripheral insulin resistance, resulting in hyperglycemia [1]. This metabolic disorder, of multifactorial etiology, is also characterized by disturbances in the metabolism of carbohydrates, fats, and proteins resulting from the aforementioned alterations [2]. Notably, in T2D, insulin resistance and fasting hyperglycemia are associated with obesity to varying degrees [3,4,5]. According to the International Diabetes Federation Atlas, in 2021, 10.5% of the adult population (aged 20–79) had diabetes, and almost half were unaware of their condition. In addition, projections for 2045 indicate that 1 in 8 adults will live with this pathology, representing a 46% increase [1].

Obesity is a chronic, progressive condition characterized by excessive body fat accumulation. This condition is a common risk factor for T2D [6]. The accumulation of adipose tissue in patients with and without T2D promotes oxidative stress, a factor that has also gained relevance in the etiology of the disease. The molecular pathways contributing to oxidative stress in obesity and/or diabetes include those related to glucose metabolism, as hyperglycemia can induce an overproduction of free radicals and reactive oxygen species, exceeding endogenous antioxidant defenses [7]. Oxidative stress is also caused by a decrease in the activity or presence of antioxidant proteins, chemical substances that restrict and delay their oxidation, such as glutathione (GSH), enzymes (catalase [CAT], glutathione peroxidase [GPx], superoxide dismutase [SOD]), micronutrients (selenium, zinc), and vitamins (C, E, and A) [8]. The comorbidities arising from pathophysiology include brain insulin resistance, neuroinflammation, and oxidative stress [9], which cause neuronal damage that ultimately leads to apoptosis [10]. Cognitive and motor regions dysfunction has been observed in T2D, characterized by reduced information-processing speed and impaired memory and executive function, which can manifest early and may be exacerbated over time [11].

In particular, the hippocampus is a sensitive region to excess nutrients, such as glucose, triglycerides, and free fatty acids (FFA), and it has also been closely implicated in regulating food intake and obesity [12,13]. The hippocampus integrates external food-sensory information and internal energy-relevant signals to evaluate when, what, and how much to eat in response to visual, olfactory, and gustatory food stimuli [13,14]. Accumulating evidence has also demonstrated that structural impairments and functional alterations in the hippocampus may be neural vulnerability factors for excess weight, food responsiveness, overeating, and the development of hyperglycemia. Cross-sectional studies have consistently found that children with obesity exhibit hippocampal atrophy that, in adulthood, is associated with impaired executive and motor functions when T2D is diagnosed [15,16]. This fact is not only associated with being overweight per se (which is often accompanied by loss of muscle mass due to metabolic diseases or neuropathy), but also with disrupted functional connectivity between the hippocampus and limbic and motor regions. However, few studies examine the early stages of obesity progression to T2D, thereby limiting the differentiation of detrimental effects on muscle-motor or brain-motor function.

The Zucker Diabetic Fatty Rat (ZDF) is a valuable animal model for studying obesity and T2D, developed in 1961 by crossing the Merck M strain and Sherman rats [17]. This model presents a mutation in the “fa” gene for the leptin receptor that prevents signaling and reduces its binding affinity. Therefore, the animals develop hyperphagia, alterations in thermogenesis, visceral adiposity, and hyperglycemia, similar to those observed in humans with obesity and T2D. Obesity develops from the third to the fifth week of life, reaching 40% of the lipid composition in their body weight by week 14, and hyperglycemia from week 12, approximately [18,19]. The ZDF strain has two phenotypes: the “Lean ZDF” (LEAN), characterized by being both lean and normoglycemic, and the “Obese ZDF” (OZDF), a homozygous (fa/fa) rat with hyperphagic behavior [20]. Therefore, this study aimed to determine the early onset of cognitive impairment and a loss of motivation (anhedonia) to engage in activities in a novel environment, both of which are associated with obesity and/or hyperglycemia, in ZDF rats.

## 2. Materials and Methods

### 2.1. Animals

Zucker Diabetic Fatty rats, 13 weeks of age (*n* = 30), were obtained from the Claude Bernard Animal Facility at the Benemerita Universidad Autonoma de Puebla (BUAP). The rats were housed under controlled environmental conditions (2 or 3 per cage), with temperatures ranging from 18 to 26 °C, humidity levels of 30–50%, a 12-h light-dark cycle, and a space of 35(w) × 55(L) × 20(h) cm, providing ad libitum access to food and water. The animals in the control group (n = 10) were fed a standard chow diet (Purina #5001), while those with the obese phenotype (without hyperglycemia; n = 10) were fed the Purina #5008 diet from weaning to week 13 of age to induce consistent development of hyperglycemia (n = 10), as recommended by Charles River Laboratories. All procedures described in this study were approved by the ethics committee CICUAL-BUAP for animal handling (100332977-UALVIEP-23/1; date 23 January 2023). The Guide for the Care and Use of Laboratory Animals of the Mexican Council for Animal Care NOM-062-ZOO-1999 was followed for each procedure described in this study. All applicable international (ARRIVE 2.0 and NHI guidelines), national, and institutional guidelines for the care and use of animals were followed to minimize possible discomfort. For the zoometry and lipid profile, n = 10 animals were used; for other determinations and analyses, n = 5 rats per group, randomly subdivided from 10 initial animals. The animals were randomly assigned using a random number generator in Excel; the procedure was performed by the veterinary staff in charge of the rats to ensure blinding.

### 2.2. Zoometric Parameters

The weight of the rats was determined using a digital scale (Torrey, model LPCR-20/40, Querétaro, Mexico), and size was measured from the base of the tail to the tip of the nose, using a flexible measuring tape. The abdominal circumference was measured at the widest point of the abdomen using a flexible measuring tape [21]. After euthanasia, for each rat, the peripancreatic, retroperitoneal, and epididymal fat was extracted and weighed individually. This procedure evaluated the distribution of visceral fat. Similarly, the weight of the muscles from the posterior limbs was recorded.

### 2.3. Biochemical Assays

#### 2.3.1. Glucose Tolerance Test (GTT)

A GTT was performed to characterize the animals’ glucose levels at 13 weeks of age. Before starting, basal glucose levels were recorded for all animals using a blood sample from their tail, obtained with a glucometer (Contour^®^ plus, Ascensia Diabetes Care Holdings AG, Basel, Switzerland). Then, the rats were fasted for 6 h with free access to water [22].

Fasting glucose was measured at the start of the test (T = 0). After the fasting period, a glucose-saline solution (2 g/kg body weight) was administered intraperitoneally, and blood glucose levels were recorded every 30 min post-injection for 2 h (at 30, 60, 90, and 120 min). Based on the final glucose curve, the animals are classified as normoglycemic (NG, ≤200 mg/dL) or hyperglycemic (HG, ≥200 mg/dL). This assay was performed between 2:00 p.m. and 4:00 p.m.

Based on both zoometric and biochemical parameters, the animals were divided into LEAN (control group, non-obese n = 10) and ZDF (obese phenotype). The ZDF group was subdivided into normoglycemic ZDF (ZDF-NG; n = 10) and hyperglycemic ZDF (ZDF-HG; n = 10) groups based on glycemia.

#### 2.3.2. Lipid Profile

Triglycerides, total cholesterol, and cholesterol in low-density lipoprotein (LDL) and high-density lipoprotein (HDL) were quantified using commercial kits in an automated spectrophotometer (A-15 BioSystems, Guadalajara, Mexico). Very low-density lipoprotein (VLDL) levels were measured using the Martin-Hopkins method [21].

#### 2.3.3. Insulin Response, Sensitivity, and Resistance Indices

The concentration of insulin was measured using ELISA immunoassay kits (Diagnostica Internacional Company, Guadalajara, Mexico) in a Stat Fax 2600 plate reader at 415 nm (WinerLab Group, Buenos Aires, Argentina). The Matsuda-DeFronzo, hepatic insulin resistance, and HOMA-β indices were calculated as previously reported [21].

### 2.4. Behavioral Tests

Following a one-week recovery period after the GTT, the animals were tested for exploratory activity behavior in the open-field test (day 1) and episodic-like memory in the novel object recognition test (NORT; days 2 and 3). All tests were video recorded for subsequent analysis. In each test, the open-field box and the objects used were cleaned after each rat was removed, removing olfactory cues.

#### 2.4.1. Open Field Test

The animals were kept in the work area with red lighting for adaptation. Later, they were placed in the open-field box (60 cm × 60 cm × 60 cm) for 10 min each. The distance traveled and movement time were recorded, and velocity was calculated for each animal [23].

#### 2.4.2. Novel Object Recognition Test (NORT)

The 3 groups (LEAN, ZDF-NG, and ZDF-HG) were subjected to the novel object recognition test to evaluate short- and long-term recognition memory [23]. NORT was assessed in the dark phase of their daily cycle, in a dark room with red light (30 lx) and isolated noise. The test consists of four stages: habituation (performed as the Open Field Test), familiarization, a short-term memory test, and a long-term memory test. Each phase lasted 6 min. Twenty-four hours after the habituation phase, the familiarization phase is carried out. Two similar, equidistant objects are placed in the box, so the animals explore identical objects. Two hours later, a short-term memory test was performed, in which one of the previously encountered objects was replaced (the positions remained the same). Twenty-four hours later, the long-term memory test is performed. Once again, the object in short-term memory is replaced with a second novel object. The time each animal spent exploring the new object (novel object recognition) was quantified. The recognition index was determined with the following equation: I_D_ = T_N_/(T_K_ + T_N_). Where $I_{D}$ is the discrimination index, $T_{N}$ is the time each animal explores the new object, and $T_{k}$ is the time each animal explores the known object [23].

### 2.5. Tissue Extraction

After the behavioral tests, the animals were anesthetized with a 0.2 mL/100 g dose of Ketamine/Xylazine, i.p. Subsequently, transcardiac perfusion with saline, followed by 4% paraformaldehyde (PFA), was performed to clear blood and preserve the brain. After brain removal, they were preserved in containers with 4% PFA or had the dorsal hippocampus dissected and frozen in liquid nitrogen, depending on the protocol they were destined to.

### 2.6. Neuronal Density

#### 2.6.1. Nissl Stain Protocol

Before sectioning, brains were placed in a 30% (*w*/*v*) sucrose solution for 3 days, and then 30 µm-thick coronal sections from the dorsal Hippocampus were obtained using a Leica cryostat (CM1510-1). The sections were mounted on gelatin-coated slides, with 4 slices per slide. Slides selected to be stained by the cresyl violet protocol, as reported previously by [23].

#### 2.6.2. Stereology Analysis

A counting grid of 100 µm × 100 µm was set inside each contour as part of a systematic random sample procedure using the optical dissector fraction (Stereo Investigator System, MicroBrightField Bioscience, Williston, Vermont, USA) on an Olympus BH2 microscope to estimate the number of neurons per region (CA1, CA3, and DG). A 50 µm × 50 µm × 50 µm counting frame with 10 µm guard zones was scanned along each of the XYZ axes using the 60× immersion objective (UPlanSAPO, Olympus Corporation, Tokyo, Japan). At least five systematically randomized sections with an area of 3600 µm^2^ were analyzed. The results were averaged and expressed as the total number of neurons per region in each hemisphere [23].

### 2.7. Hippocampal Redox Balance Analyses

Hippocampal tissue (n = 6 per group) was used to evaluate redox balance. The tissues were homogenized and centrifuged at 18,000× *g* for 30 min at 4 °C. The supernatant was used to assess the following parameters:

#### 2.7.1. Reactive Oxygen Species (ROS)

The levels of ROS were measured using a 2′,7′-dichlorofluorescein diacetate (DCFH-DA) assay. Frozen hippocampus samples were homogenized in PBS 1× and then centrifuged. 50 µL of the supernatant was incubated with DCFH-DA in the dark at 37 °C for 20 min. Intracellular ROS oxidizes the non-fluorescent DCFH-DA to form the fluorescent compound 2′,7′-dichlorofluorescein (DCF). Fluorescence intensity was measured at T = 0, 3, and 5 min with excitation at 480 nm and emission at 530 nm using a SmartSpec 3000 spectrophotometer (Bio-Rad, Hercules, CA, USA). ROS levels were expressed as nanomol (nM) normalized with total protein content [23].

#### 2.7.2. Nitrites

Nitrite concentration, as an indicator of nitric oxide (NO) production, was quantified using the Griess reagent [24]. Briefly, 100 µL of supernatant was mixed with 100 µL of Griess reagent (0.1% N-(1-naphthyl) ethylenediamine dihydrochloride and 1.32% sulfanilamide in 60% acetic acid) and 80 µL of distilled water. The absorbance was measured at 540 nm using a SmartSpec 3000 spectrophotometer (Bio-Rad, Hercules, CA, USA). Nitrite concentration was calculated using a sodium nitrite standard curve (1 to 10 µM) and expressed as µM/mg of protein [23].

#### 2.7.3. Lipoperoxidation (MDA Levels)

Lipid peroxidation was assessed by measuring malondialdehyde (MDA). 100 µL of supernatant was mixed with 650 µL of a solution containing 1-methyl-2-phenylindole diluted in a mixture of acetonitrile and methanol [23]. After vigorous shaking, 150 µL of methanesulfonic acid was added to the mixture, which was then incubated for 40 min at 45 °C. Once the mixture cooled, it was centrifuged at 3000 rpm for 15 min, and the reading was performed using a SmartSpec 3000 spectrophotometer (Bio-Rad, Hercules, CA, USA) at 586 nm. The concentration of both markers is calculated using the calibration curve. The MDA concentrations were calculated using the 1,1,3,3,3-pentamethoxypropane standard curve [23].

#### 2.7.4. Evaluation of Antioxidant Enzyme Levels

The glutathione peroxidase (GPx) activity reduces hydroperoxides to form tert-butyl hydrogen peroxide, which in turn leads to the oxidation of the enzyme. Therefore, the decrease in NADPH is directly proportional to GPx activity, which was measured spectrophotometrically at 340 nm using a PerkinElmer Lambda EZ-150 spectrophotometer and expressed in micromoles per minute per milligram of protein. The medium contained 2 mM GSH, 0.15 U/mL GR, 0.4 mM sodium azide, 10 mM tert-butyl hydroperoxide, 0.5 mM 0.1 mM NADPH, and 26 μL of sample [24].

Glutathione s-Transferase (GST) activity is based on the conjugation of CDNB (1-chloro-2,4-dinitrobenzene) to reduced glutathione (GSH). The working solution consisted of 33 mM HEPES buffer at pH 7.5, 1.5 mM GSH, 1-chloro-2-dinitrobenzene (CDNB), and water, for a total volume of 1 mL. The conjugation of GSH and CDNB activates Transferase, and its activity was monitored for 3 min by spectrophotometry at 340 nm, expressed as micromoles per minute per milligram of protein [24].

The activity of the enzyme glutathione reductase (GR) was determined following the oxidation of NADPH in the presence of oxidized glutathione, GSSG. The resulting GSH reacts with DTNB, leading to an increase in absorbance at 412 nm. The reaction mixture consisted of 0.1 M phosphate buffer (pH 7.5), 1 mM EDTA, 2 mM GSSG, and 3 mM DTNB. The reaction begins after the addition of 2 mM NADPH [25]. The catalase activity (CAT; U min^−1^/mg of protein) was quantified using the Aebi method [24].

### 2.8. Statistical Analysis

A Shapiro–Wilk normality test was performed to verify that the data sets are typically distributed. The results were expressed as the mean ± SEM for all experiments. Zoometric parameters, open field test data, NORT data, neuron density results, and oxidative stress levels were analyzed using a two-way ANOVA followed by a Bonferroni post hoc test. A two-way repeated-measures ANOVA followed by Bonferroni’s post hoc test was used to evaluate the effects of obesity and hyperglycemia on all parameters analyzed. Their interaction was assessed using the F-statistic, calculated as the mean square for the time factor divided by the residual mean square, with the degrees of freedom used to determine the F-statistic distribution. A *p*-value of less than 0.05 was considered significant for all statistical tests. We tested the correlation between variables (weight and hyperglycemia) using a two-tailed Pearson test (distance, velocity, behavioral tests, and histological data). The statistics were performed using GraphPad Prism 8.0 (GraphPad Software Inc., Boston, MA, USA).

## 3. Results

### 3.1. Zoometric Parameters

As part of characterizing the animal model, parameters such as weight, abdominal length, fat, and muscle were measured (Table 1). The ZDF groups showed differences in weight compared with the LEAN control group, but hyperglycemia showed a low interaction (F_(2,13)_ = 4.33; *p* < 0.0001). The length of the animals did not differ between groups. The abdominal circumference was the major contributor in the ZDF groups, and glycemia interacted with variables (F_(2,13)_ = 62.91; *p* < 0.0001), denoting central obesity. Fat content was higher in the ZDF group: retroperitoneal (F_(2,13)_ = 33.69; *p* < 0.0001), epididymal (F_(2,13)_ = 35.40; *p* < 0.0001), and peripancreatic (F_(2,13)_ = 27.09; *p* < 0.0001). However, muscle weight did not differ between groups.

### 3.2. Lipid Profile

The lipid profile of the analyzed groups showed hyperlipidemia in the ZDF groups compared with the LEAN control group (Table 2). The levels of cholesterol in the ZDF groups are significantly higher (205.6 and 276.8 mg/dL) than the LEAN group (113 mg/dL; F_(2,18)_ = 32.59; *p* < 0.0001). The VLDL level in the ZDF-NG (66 mg/dL) and ZDF-HG group (38 mg/dL) was higher than that of the LEAN control (14 mg/dL; F_(2,18)_ = 13.32; *p* = 0.0003). The HDL level showed differences between the ZDF groups: it decreased by 27% in the ZDF-NG group and increased by 22.4% in the ZDF-HG group, with a low glucose interaction (F_(2,18)_ = 5.02; *p* = 0.0185). LDL levels increased by 70.5% (ZDF-NG) and 92.6% (ZDF-HG), with a low variable interaction (F_(2,18)_ = 7.043; *p* = 0.0055). Finally, triglyceride levels increased by 385.8% (ZDF-NG) and 267.8% (ZDF-HG), with a mild variable interaction (F_(2,18)_ = 10.42; *p* = 0.0011).

### 3.3. Glucose Tolerance, Insulin Response, and Resistance Indices

**Figure 1:**
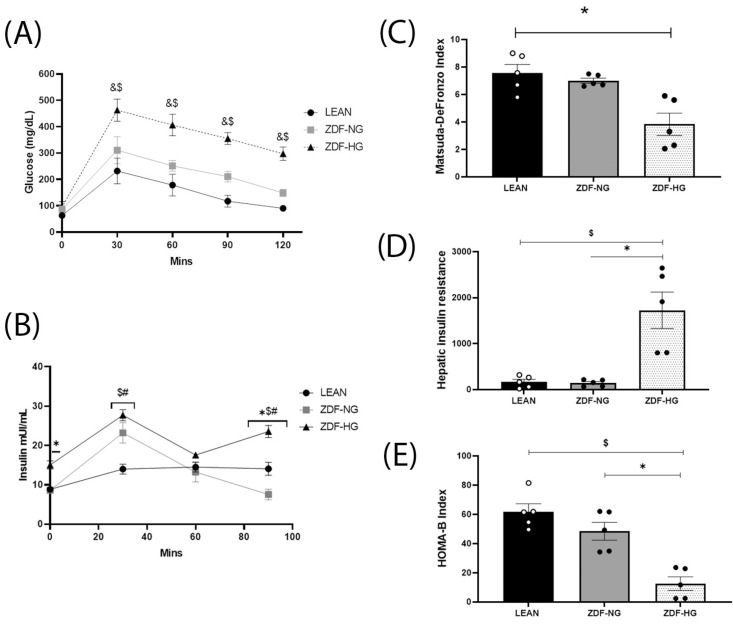
Glucose tolerance, insulin response, and insulin indices. Glucose tolerance curve (**A**). Insulin curve (**B**). Matsuda-DeFronzo index (**C**). Hepatic insulin resistance index (**D**). HOMA-β index (**E**). The results shown are the average of ten different experiments ± SEM (n = 5). A Two-way ANOVA and Bonferroni post hoc test were performed with a significance level set at *p* ≤ 0.05. Data from the ZDF-NG and ZDF-HG groups were compared with the Lean control group. Significant respect to LEAN group * *p* < 0.05, ^$^ *p* < 0.005, ^#^ *p* < 0.0005, ^&^ *p* < 0.0001.

Glucose tolerance tests were performed to validate the hyperglycemia status of the study groups before conducting the behavioral trials (Figure 1A). The ZDF-HG group showed fasting glucose levels higher than those of the ZDF-NG and LEAN groups (95.4 mg/dL, 86.6 mg/dL, and 63 mg/dL, respectively; F_(2,59)_ = 49.89; *p* < 0.0001). At 30 min, glucose in the LEAN group was 231.8 mg/dL, in the ZDF-NG group was 310.6 mg/dL, and in the ZDF-HG group it was 463 mg/dL (F_(2,59)_ = 53.91; *p* < 0.0001). At 60 min, glucose in the LEAN group was 178.4 mg/dL, in the ZDF-NG group was 251.4 mg/dL, and in the ZDF-HG group it was 406.3 mg/dL (F_(2,59)_ = 58.41; *p* < 0.0001). At 90 min, glucose in the LEAN group was 117.4 mg/dL, in the ZDF-NG group was 210.8 mg/dL, and in the ZDF-HG group it was 355 mg/dL (F_(2,59)_ = 49.73.91; *p* < 0.0001). At 120 min, glucose in the LEAN group was 90.2 mg/dL, in the ZDF-NG group was 148.4 mg/dL, and in the ZDF-HG group it was 297.2 mg/dL (F_(2,59)_ = 83.22; *p* < 0.0001). The interaction between variables strongly suggests that obesity is associated with glucose intolerance and the development of hyperglycemia.

Fasting hyperinsulinemia was only present in the ZDF-HG (15.1 mUI/mL), with low, non-significant interaction (F_(6,24)_ = 6.303; *p* = 0.0004). However, at 30 min post glucose load, both ZDF-NG and ZDF-HG groups presented hyperinsulinemia (22.9 and 27.5 mUI/mL) compared with the LEAN group (14 mUI/mL; F_(6,24)_ = 27.58; *p* < 0.0001), suggesting that normoglycemic obesity is a transitory stage, because variables showed a high interaction. At 60 min post glucose load, there is no difference between groups. However, at 90 min of analysis, insulin concentration decreased by 41.8% in the ZDF-NG group compared with the LEAN group, whereas it increased by 62.1% in the ZDF-HG group (Figure 1B), confirming a transitory stage from normo- to hyperglycemia (F_(6,24)_ = 34.89; *p* < 0.0001).

Additionally, insulin sensitivity was evaluated using the Matsuda-DeFronzo index, which showed a significant 46.7% decrease in the ZDF-HG group. The interaction indicates that only the hyperglycemia stage influences insulin sensitivity loss (F_(2,7)_ = 6.229; *p* = 0.0279; Figure 1C). To confirm this finding, the ZDF-HG group showed a significantly increased hepatic insulin resistance index (F_(2,7)_ = 11.96; *p* = 0.0055; Figure 1D). Finally, β-cell function was evaluated using the HOMA-β index, results showing a loss of β-cell function associated with obesity and exacerbated by hyperglycemia (F_(2,7)_ = 12.58; *p* = 0.0048; Figure 1E).

### 3.4. Behavioral Tests

#### 3.4.1. Motor Activity

**Figure 2:**
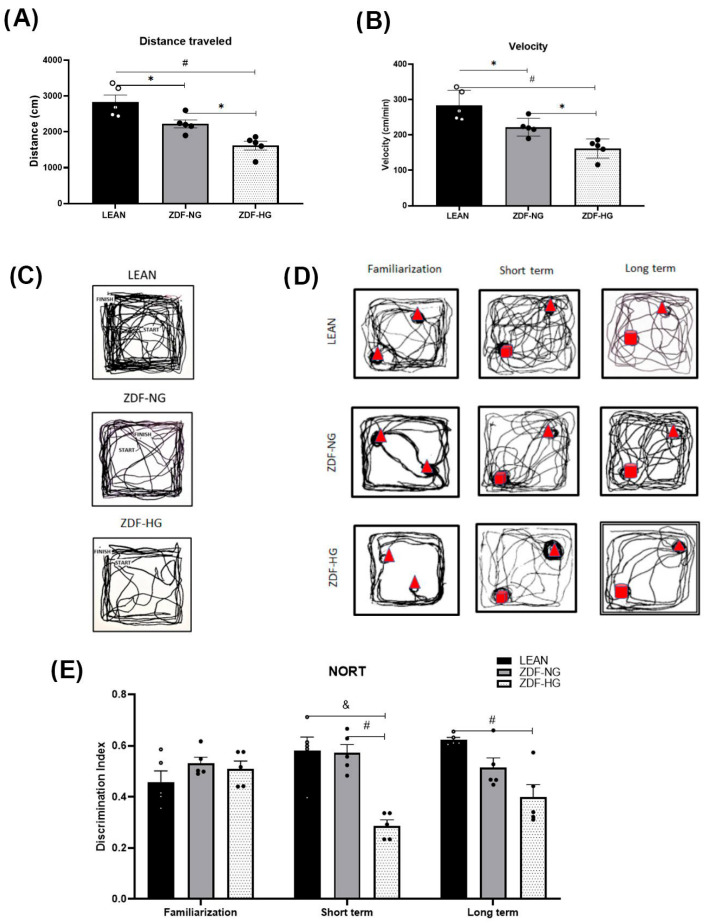
The ZDF group (normo- and hyperglycemic) shows less activity in the new environment than the LEAN control. (**A**) Distance (cm). (**B**) Displacement velocity expressed in cm/min. (**C**) Representative trajectories for each group in the open field test. (**D**) Representative trajectories of NORT for each group, the familiar object represented as a triangle, and the novel objects as a cube and a cylinder for short-term memory and long-term memory. (**E**) Evaluation of short- and long-term memory (NORT). The results shown are the average of ten different experiments ± SEM (n = 5). A Two-way ANOVA and Bonferroni post hoc test were performed with a significance level set at *p* ≤ 0.05. Data from the ZDF-NG and ZDF-HG groups were compared with the Lean control group. Significant respect to LEAN group * *p* < 0.05, ^#^ *p* < 0.0005, ^&^ *p* < 0.0001.

The ZDF groups travelled a shorter distance slowly, not weight-dependent, but exacerbated by hyperglycemia (Figure 2A–C), in the open field test compared with the LEAN control group (F_(2,12)_ = 17.49; *p* = 0.0003). Therefore, although obesity is a common factor in ZDF, the hyperglycemic group travels a shorter distance than the normoglycemic group (LEAN vs. ZDF-NG, *p* = 0.0284; LEAN vs. ZDF-HG, *p* = 0.0002; and ZDF-NG vs. ZDF-HG, *p* = 0.0315).

#### 3.4.2. Novel Object Recognition Test (NORT)

The familiarization phase of this test does not show differences between groups (F_(2,34)_ = 0.4891; *p* = 0.6174; Figure 2D), but in the short- (F_(2,34)_ = 6.555; *p* = 0.0005), and long-term memory phases (F_(2,34)_ = 14.38; *p* < 0.0001), the increase in the discrimination index evidences an adequate performance in the LEAN and ZDF-NG groups; however, the ZDF-HG group decreases the discrimination index in both types of memory in relation to the familiarization phase, evidencing alterations in the long term potentiation and hyperglycemia as detriment condition (Figure 2E).

### 3.5. Neuronal Density of Hippocampus

**Figure 3:**
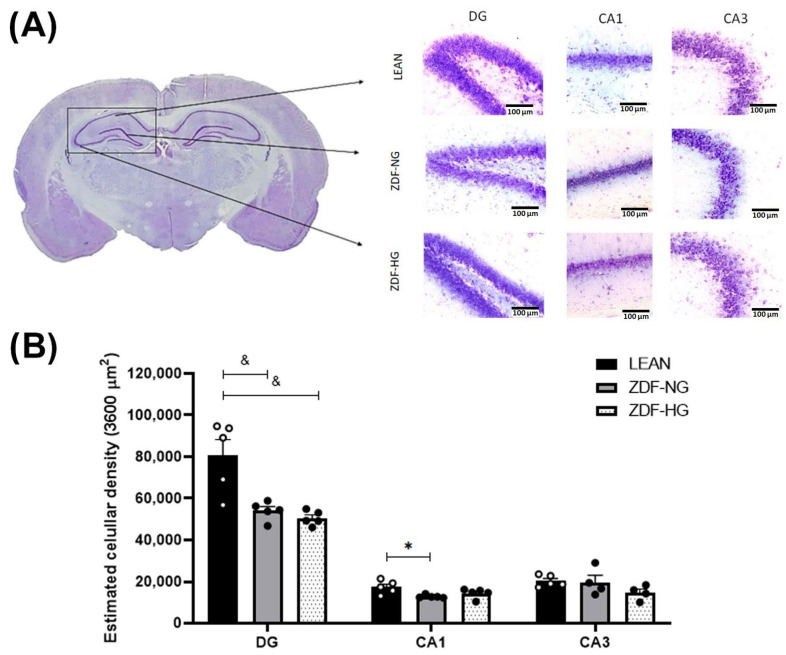
Representative coronal slice image showing rat hippocampal DG, CA1, and CA3. (**A**) Hippocampal region at 20× magnification for the analyzed groups, 100 µm calibration bars. (**B**) Estimated cell population in a 3600 µm^2^. The results shown are the average of ten different experiments ± SEM (n = 5). A Two-way ANOVA and Bonferroni post hoc test were performed with a significance level set at *p* ≤ 0.05. Data from the ZDF-NG and ZDF-HG groups were compared with the Lean control group. Significant respect to LEAN group * *p* < 0.05, ^&^ *p* < 0.0001.

Neuronal density, determined by stereological analysis, was analyzed in hippocampal DG, CA1, and CA3 regions (Figure 3A). The results showed a decrease in the number of neurons in the hippocampal DG in the ZDF groups compared to the LEAN control group, indicating that obesity, but not hyperglycemia, is responsible (F_(4,34)_ = 7.921; *p* = 0.0001). The number of neurons in the hippocampal CA1 region only diminished in the ZDF-NG (F_(2,34)_ = 223.5; *p* < 0.0001). The number of neurons in the hippocampal CA3 did not differ between groups (Figure 3B).

### 3.6. Hippocampal Redox Balance

**Figure 4:**
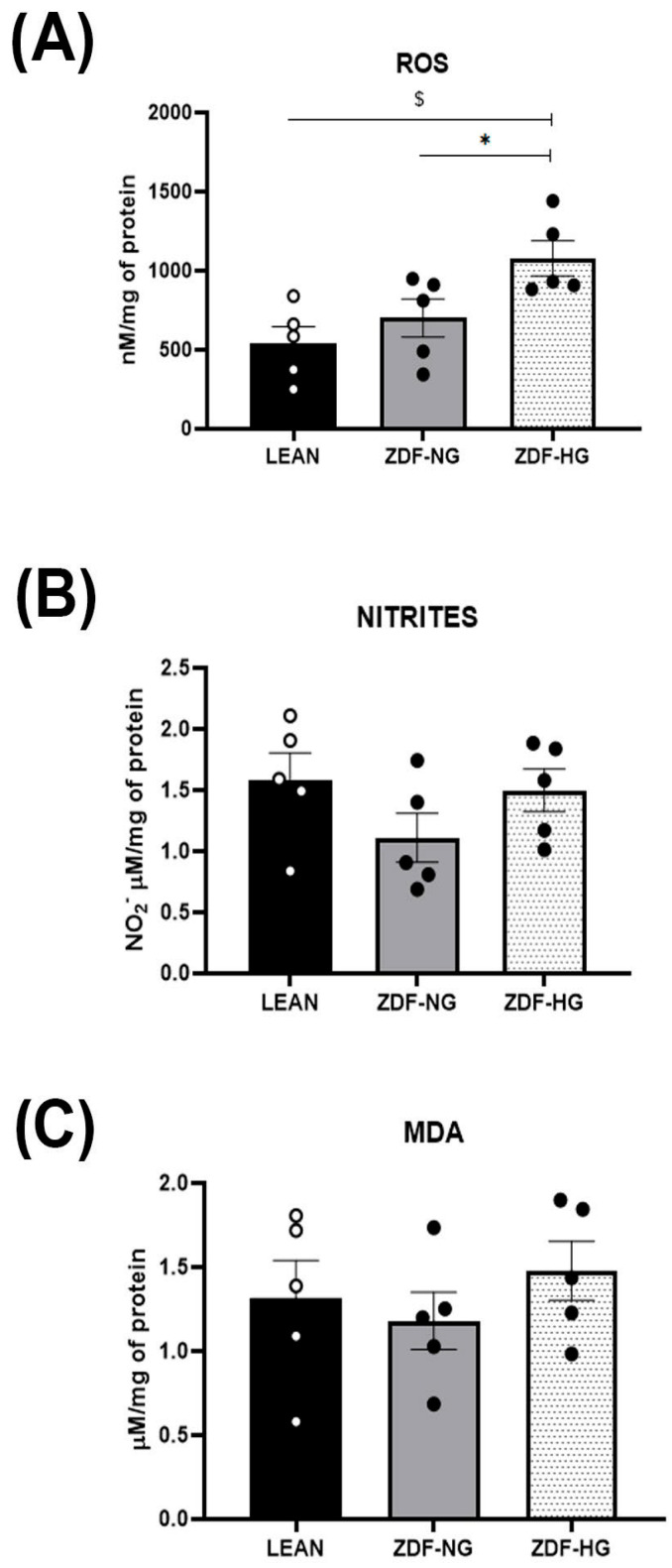
Oxidative stress. (**A**) Reactive oxygen species (ROS). (**B**) Nitrites. (**C**) Lipoperoxidation (MDA). The results shown are the average of ten different experiments ± SEM (n = 5). A Two-way ANOVA and Bonferroni post hoc test were performed with a significance level set at *p* ≤ 0.05. Data from the ZDF-NG and ZDF-HG groups were compared versus the Lean control group. Significant respect to LEAN group * *p* < 0.05, ^$^ *p* < 0.005.

**Figure 5:**
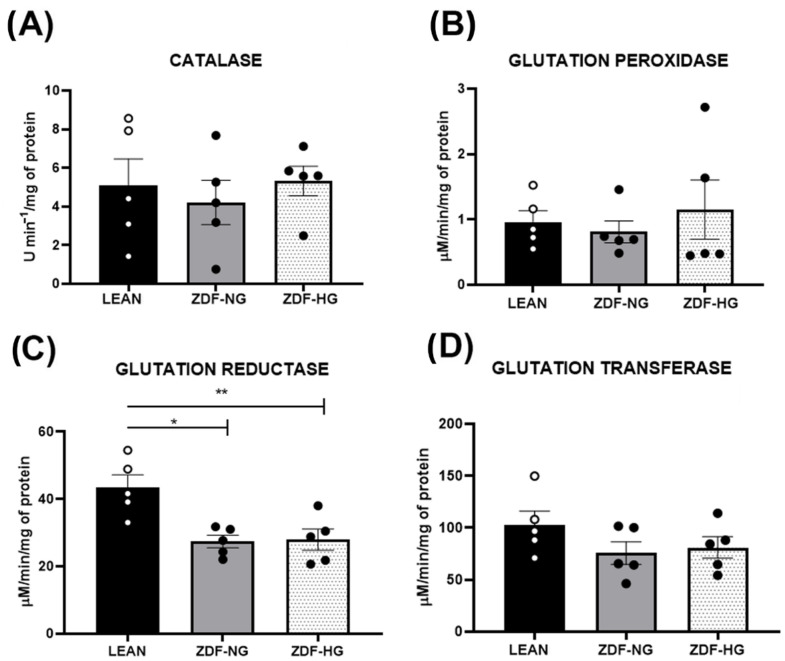
Antioxidative enzymes. (**A**) Catalase activity. (**B**) Glutathione peroxidase. (**C**) Glutathione reductase. (**D**) Glutathione transferase. The results shown are the average of ten different experiments ± SEM (n = 5). A Two-way ANOVA and Bonferroni post hoc test were performed with a significance level set at *p* ≤ 0.05. Data from the ZDF-NG and ZDF-HG groups were compared with the Lean control group. Significant respect to LEAN group * *p* < 0.05, ** *p* < 0.01.

Reactive oxygen species (ROS) levels were significantly higher in the hyperglycemic group than in the normoglycemic and control groups (F_(2,15)_ = 8.0; *p* = 0.0030; Figure 4A). Interaction analysis indicates that both obesity and hyperglycemia contribute to ROS increase. Nitrite levels and lipoperoxidation did not differ between groups (F_(2,15)_= 2.323; *p* = 0.1322 and F_(2,15)_ = 0.8427; *p* = 0.4499; Figure 4B,C). The activity of antioxidant enzymes: catalase, glutathione peroxidase, and transferase did not show differences between groups (Figure 5A,B,D). However, glutathione reductase levels decreased in both obese groups, without an interaction with hyperglycemia (F_(2,13)_ = 1.179; *p* = 0.3384; Figure 5C).

### 3.7. Influence of Weight and Hyperglycemia, Correlation Analysis

**Figure 6:**
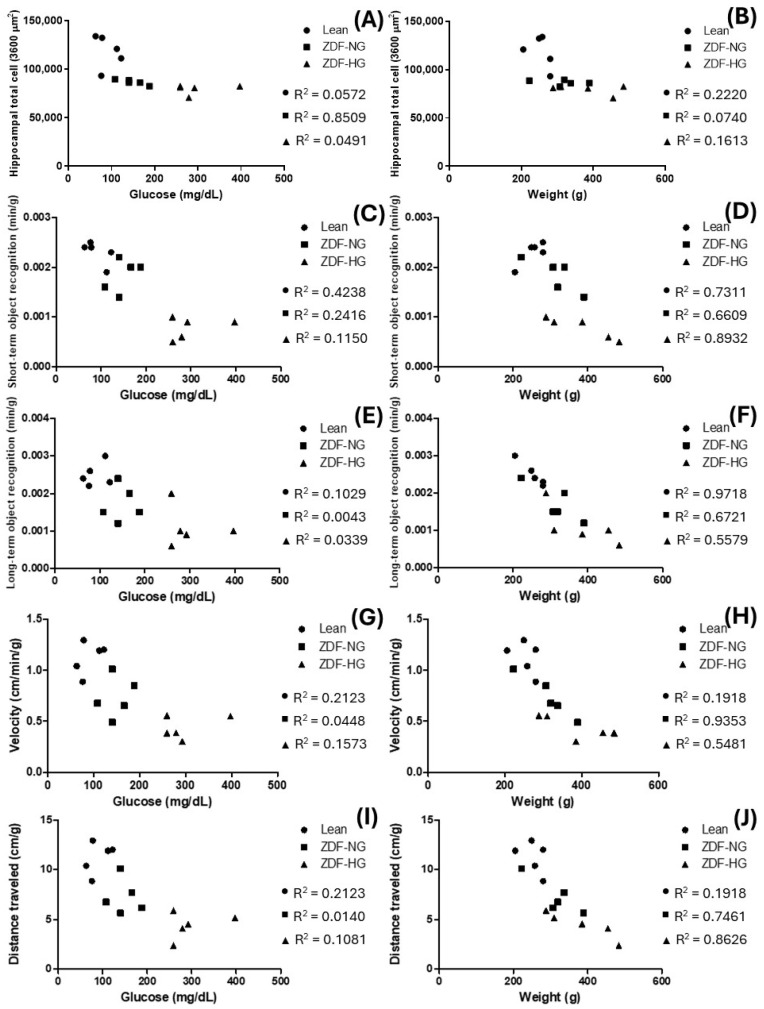
Spearman’s correlation between obesity and glycemia on motor motivation hippocampal-associated. (**A**) Hippocampal total cell and glycemia correlation; (**B**) Hippocampal total cell and body weight correlation; (**C**) Time of short- term object recognition and glycemia correlation; (**D**) Time of short- term object recognition and body weight correlation; (**E**) Time of long- term object recognition and glycemia correlation; (**F**) Time of short- term object recognition and body weight correlation; (**G**) Velocity of travel distance and glycemia correlation; (**H**) Velocity of travel distance and body weight correlation; (**I**) Distance traveled and glycemia correlation; (**J**) Distance traveled and body weight correlation. The figures show the determination coefficient (R^2^) for each group: the LEAN control group (•), the ZDF-NG group (■), and the ZDF-HG group (▲). The correlation between variables (weight and glycemia) was tested using a two-tailed Pearson test at *p* < 0.05.

To determine whether weight or hyperglycemia influences hippocampal deterioration, we performed a correlation analysis, in which we first normalized the results to grams of rat to avoid redundant analyses and bias, except for the number of total hippocampal cells (Figure 6A,B). The results showed a strong negative correlation between hyperglycemia and the total number of hippocampal cells (Pearson r = −0.2391 [LEAN]; −0.9224 [ZDF-NG]; and 0.2216 [ZDF-HG]), with R^2^ values of 0.0572, 0.8509, and 0.0491, respectively. Only the ZDF-NG was statistically significant (*p* = 0.0256). However, when we analyzed the influence of weight on the total number of hippocampal cells, the results showed a Pearson r = −0.4712 [Lean]; −0.2720 [ZDF-NG]; and −0.4017 [ZDF-HG], a mild to moderate negative correlation with an R^2^ of 0.2220, 0.07399, and 0.1613, respectively, without statistical significance (*p* = 0.4230, 0.6580, and 0.5027).

The influence of weight or hyperglycemia on the time spent exploring novel objects was also examined. The short-term results in the Lean group showed a negative correlation with glycemia (Pearson r = −0.6510), with R^2^ = 0.4238 and *p* = 0.2341, suggesting that even small fluctuations in glucose toward the upper limit can reduce curiosity or exploration efficiency. The inverse correlation in the ZDF-NG and ZDF-HG groups (Pearson r = 0.4915 and 0.3392) suggests a change in the behavioral strategy, with R^2^ of 0.2416 and 0.1150, respectively, not significant (*p* = 0.4004 and 0.5766). Conversely, the weight correlation shows a positive correlation (Pearson r = 0.8550), with R^2^ = 0.7311 and *p* = 0.0648, indicating that animals with normal weight exhibited more active exploration. However, being overweight reverses the correlation, and hyperglycemia exacerbates the exploration deficit (Pearson r = −0.8130 and −0.9451; R^2^ = 0.6609 and 0.8932), which was significant in ZDF-HG groups (*p* = 0.0153; Figure 6C,D). Meanwhile, the long-term exploration of novel objects, glycemia was very low correlated in all analyzed groups (Pearson r = 0.3208 [Lean]; 0.0653 [ZDF-NG]; and −0.1840 [ZDF-HG]), with R^2^ values of 0.1029, 0.0043, and 0.0339, respectively, without significance (*p* = 0.5986, 0.9169, and 0.7671). However, the weight presented a strong negative correlation in the study groups (Pearson r = −0.9858 [LEAN]; −0.8198 [ZDF-NG]; and −0.7469 [ZDF-HG]), with R^2^ values of 0.9718, 0.6721, and 0.5579, respectively; although only the Lean group was significant (*p* = 0.0020), confirming the importance of body weight on information acquisition (Figure 6E,F).

Body weight appears to be a decisive factor in hippocampal deterioration and the loss of the impulse to acquire new information; however, this is attributable solely to excess weight. Therefore, we analyzed the correlation between the motor activity and velocity of biomodels with our main variables, weight and hyperglycemia. The results showed no correlation between blood glucose and distance traveled, indicating that glycemia does not affect the rat’s physical ability to move, even in obese and obese-hyperglycemic ZDF rats (Pearson r = 0.4608 [LEAN]; −0.1183 [ZDF-NG]; and 0.3287 [ZDF-HG]), with R^2^ values of 0.2123, 0.0140, and 0.1081, respectively, without significance (*p* = 0.4348, 0.8497, and 0.5891). Conversely, the body weight showed a negative correlation, which was stronger and significant in the ZDF-HG (*p* = 0.0226), Pearson r = −0.4379 (LEAN); −0.8638 (ZDF-NG); and −0.9288 (ZDF-HG), with R^2^ values of 0.1918, 0.7461, and 0.8626 (Figure 6G,H). Similar to the distance traveled, the travel velocity did not show statistical significance (Pearson r = 0.4608 [LEAN]; 0.2117 [ZDF-NG]; and 0.3966 [ZDF-HG]), with R^2^ values of 0.2123, 0.0448, and 0.1573, respectively, without significance (*p* = 0.4348, 0.7325, and 0.5086), thereby rats’ displacement is comparable between groups. Meanwhile, the correlation analysis confirmed that body weight is the most critical factor that limits rats’ neurobehavior, a negative correlation with a Pearson r of −0.4379 (LEAN, R^2^ = 0.1918); −0.9671 (ZDF-NG, R^2^ = 0.9353); and −0.7403 (ZDF-HG, R^2^ = 0.5481), significantly only for the ZDF-NG group (*p* = 0.0071; Figure 6I,J).

## 4. Discussion

The ZDF rat is a spontaneous, intrinsic animal model that does not aim to mimic T2D but rather to address specific questions and aspects of it. Its characteristic, the leptin receptor mutation, induces hyperphagia, leading to obesity and hyperglycemia. Therefore, the study aimed to determine the early onset of cognitive impairment and a loss of motivation (anhedonia) to engage in activities in a novel environment associated with obesity and/or hyperglycemia in ZDF rats. Obesity and T2D complications lead to cognitive impairment [25], and evidence indicates that disturbances in glucose and lipid metabolism affect brain function [26].

We subdivided the obese group into normoglycemic (ZDF-NG) and hyperglycemic (ZDF-HG) based on postprandial glycemia to evaluate the onset of hyperglycemia associated with obesity, and we contrasted these results with a LEAN control group (Table 1 and Figure 1). This corroborates what has been reported in the literature about the ZDF strain, since, as mentioned above in the background, Wang et al. [19] distinguish between hyperglycemic and normoglycemic groups. However, the parameters of total cholesterol, triglycerides, HDL, LDL, and VLDL demonstrated dyslipidemia in both groups, ZDF-NG and ZDF-HG (Table 2). Obesity is a consequence of a positive energy balance, leading to an increase in fat mass, particularly abdominal obesity, characterized by the deposition of abdominal fat, which has been a prominent health hazard associated with metabolic syndrome and T2D [27].

Particularly in T2D, hyperglycemia is caused by chronic impairment of insulin signaling, decreased insulin sensitivity, or the development of insulin resistance (IR), as stated by Li et al. [28], and observed in our results. Insulin resistance is a hallmark of many metabolic diseases; however, although almost all tissues possess insulin receptors and signaling pathways associated with the hormone (metabolic and mitogenic), not all develop IR simultaneously or with the same features [21]. Insulin resistance is the inability of tissues to respond to the hormone, so higher insulin concentrations are required to maintain normal function, as observed in our results (Figure 1B,C). In particular, in the liver, insulin activity maintains glucose and lipid homeostasis by suppressing gluconeogenesis, promoting glycogen synthesis, and stimulating lipogenesis [29]. Hepatic IR, as shown (Figure 1C), promotes fasting dysglycemia and dyslipidemia, with increases in triglycerides, LDL, and VLDL, while simultaneously diminishing HDL, as we observed (Table 2).

The liver–adipose axis is central to the management of carbohydrates and lipids, in which insulin signaling promotes the biotransformation of excess carbohydrates into lipids via *de novo* lipogenesis (DNL). In both tissues, this process is regulated by transcriptional factors, such as sterol regulatory element-binding protein 1 and carbohydrate response element-binding protein [29]. Additionally, DNL is redundant, as it synthesizes and recycles fatty acids (FAs) released from adipose tissue during fasting. In the liver, FAs converted to triglycerides are stored in lipid droplets or released to the bloodstream as VLDL [21,30]. Triglycerides in the lipoproteins are carried and stored in adipose tissue, where insulin coordinates this process [30,31]. Adipose tissue stores lipids and simultaneously secretes adipokines (such as leptin and adiponectin) that play a vital role in regulating food intake and energy expenditure [32]. Because ZDF rats have a mutation in the leptin receptor, they lack satiety; therefore, they develop lipid overstorage, adipose expansion, and obesity, as well as hyperglycemia over time, as observed in our results.

Altered lipid and glucose homeostasis have been shown to lead to memory loss [9,23]. In our study, short- and long-term recognition memory were evaluated, including possible hippocampal damage (Figure 2C–E). The novel object recognition test (NORT) showed a decrease in the discrimination index in the ZDF-HG group, suggesting impairment of both short- and long-term memory. These results partially coincide with those reported by Jolivalt et al. [33], who demonstrated alterations in short-term memory in this same animal model at 16 weeks of sustained hyperglycemia. In addition, hyperglycemia not only affects memory processes but also obesity, which is closely related to this cognitive process. Other studies with T1D and T2D models have shown a relationship between these memory deficits and reduced expression of synaptic proteins [34]. Lizarbe et al. [35] demonstrated changes in the expression of presynaptic proteins, such as synaptophysin and syntaxin-1, in the hippocampus, cortex, and hypothalamus of mice exposed to hypercaloric diets. Furthermore, Treviño et al. [36] demonstrated in an animal model of metabolic syndrome that consuming a high-calorie diet and hyperglycemia impair recognition memory, reduce synaptic plasticity, and lower synaptophysin immunoreactivity in the hippocampus, leading to neurodegeneration that affects learning and memory.

In the hippocampus, learning and memory depend on adequate early long-term potentiation (eLTP). Studies in obese and T2D animal models have shown alterations in eLTP and synaptic plasticity in the cerebral cortex and hippocampus, leading to memory deficits [37,38,39]. In addition, in recent years, it has been hypothesized that metabolic dysfunction is associated with impaired insulin signaling in hippocampal neurons, dysregulating neurotransmitters involved in memory, such as glutamate, noradrenaline, and acetylcholine [39,40]. Sustained hyperglycemia and dyslipidemia induce the formation of advanced glycation end products and lipid peroxidation via oxidative stress [41]. Studies on metabolic syndrome in Wistar rats show increased ROS and lipid peroxidation products in the hippocampus, along with decreased activity of the antioxidant enzymes SOD and CAT, confirming the role of oxidative stress in the structural and functional damage to the hippocampus [23]. Moreover, Raza et al. [42] reported oxidative stress in ZDF rats at 20 weeks, evidenced by a significant increase in ROS in the whole brain. Our results confirmed that in obese ZDF groups (with and without hyperglycemia), hippocampal ROS increased, while GR activity decreased. Although, all other parameters analyzed in the redox profile, such as nitrites, MDA, as well as CAT, GPx, and GST activity showed no differences between groups, suggesting that, in onset stage the oxidative stress did not trigger hippocampal damage (Figure 4 and Figure 5), like reported in 9-month-old ZDF-DIA (diabetic ZDF) rats [43] and in brain of ZDF rats at 17 weeks of age [44].

However, our histological evaluation of the hippocampus by stereology, a decrease in the neuronal density (Figure 3), mainly in the dentate gyrus in the ZDF-HG group and CA1 in the ZDF-NG, has been shown, which has not been reported before, so this could explain recognition memory impairment and also indicate that obesity and hyperglycemic conditions induce damage to these regions. Studies in Zucker rats have reported alterations in the neurons of the hippocampal dentate gyrus, specifically a decrease in dendritic length and a reduction in dendritic spine density with obesity and hyperglycemia [44]. Since oxidative stress does not appear to be the inducing factor in neural death, inflammation may be the cause, although this must be investigated in future studies on the ZDF strain, as it is well known in other rat strains with obesity or hyperglycemia [23,37]. Obesity, in turn, contributes to the inflammatory state by activating NF-κB, which induces the expression of inflammatory cytokines [45]. In addition, impaired recognition memory has been associated with elevated levels of proinflammatory cytokines (IL-1β, TNF-α, and IL-6). This suggests a possible correlation between inflammation caused by T2D, metabolic imbalance, and memory impairment [46], which could explain how hippocampal neurodegeneration induced loss of short- and long-term memory in ZDF-HG rats.

The hippocampus, traditionally known for memory, is also a critical component of the neural circuitry regulating motivation and impulsivity, with specific functions localized to its different subregions, primarily the ventral hippocampus (vHP) [47]. The vHP-cingulo-parietal network, involved in cognitive control and executive functioning, may play a role in the susceptibility of ZDF obese and hyperglycemic rats to risk-taking when its connectivity with the hippocampus is altered. The vHP also regulates impulsive action through specific pathways. A neural pathway from the vHP to the nucleus accumbens (NAc) and the medial prefrontal cortex is crucial for inhibiting impulsive responses [48]. Dysfunction in these circuits contributes to conditions characterized by severe changes in impulsivity, including alterations in motor motivation. The primary output regions of the hippocampus that are projected to the NAc are the CA1 and the subiculum subregions of both the dorsal and ventral hippocampus [48]. However, these neural networks must be studied in depth to understand their role in the context of obesity and hyperglycemia.

In this way, the present study has shown a decrease in the motor activity of both normoglycemic and hyperglycemic ZDF groups (Figure 2A,B), as analyzed by the open-field test, which is consistent with Li et al. [26], who reported diminished horizontal and vertical movement of ZDF rats at 14 weeks of age, assessed with the same test. Some reports have shown that in the obese Zucker rat, there is an increase in skeletal muscle protein degradation [49], as well as a reduction in muscle mass compared to lean Zucker rats and alterations in the muscle adaptation capacity [50,51]; this means that the muscles do not present an adequate response to the increased load, greater weight. However, our results showed that muscle mass did not differ between groups. Additionally, our results show differences in motor activity within the glycemic subgroups of the obese group (ZDF-NG vs. ZDF-HG). Therefore, it is possible to argue that the decrease in motor activity may be due to alterations in the neural network of circuitry regulating motivation and impulsivity.

To determine whether obesity, hyperglycemia, or both are associated with a loss of motor motivation, we performed a correlation analysis with both variables as predictors. First, we analyzed the correlation between hippocampal total cells and glycemia; our results reveal an interesting dichotomy in hippocampal integrity along the progression of metabolic state. In normoglycemic obese rats, we observed a robust negative correlation, suggesting that the hippocampus is susceptible to early metabolic alterations associated with obesity. Surprisingly, this correlation is lost in the chronic hyperglycemic state, suggesting maximal cell damage or the activation of nonlinear cell death mechanisms in advanced stages of hyperglycemia (Figure 6A). However, the damage to the hippocampal cells is not entirely dependent on obesity, because of the high variability of the results before hyperglycemia, confirming that there was sufficient cell damage before hyperglycemia (Figure 6B). Then we correlated the time of exploration in the short and long-term against both interest variables. Correlation analysis revealed that body weight differentially impacts short-term recognition memory depending on metabolic status. While lean animals showed a positive trend, obese groups exhibited a negative association. Notably, in the hyperglycemic obese group, body weight significantly predicted cognitive impairment, suggesting that the progression of obesity under hyperglycemic conditions is a critical determinant of hippocampal dysfunction (Figure 6C,D).

Additionally, body weight is a critical factor of long-term exploratory behavior in the lean control group, showing an almost perfect negative correlation. However, this linear association progressively weakens in the normoglycemic and hyperglycemic obese groups, suggesting that the pathophysiology of obesity and diabetes introduces additional variables that alter the normal allometric relationship between weight and cognitive-behavioral activity (Figure 6E,F). To rule out the possibility that recognition memory deficiencies influenced locomotor activity, the correlation between blood glucose levels and total distance traveled was analyzed. No significant association was found in any of the groups evaluated. These results confirm that metabolic status and glycemic load did not compromise the motor ability of the experimental subjects during the behavioral tests (Figure 6G,H).

Finally, the analysis of movement speed showed no significant correlation with baseline glucose levels in any of the experimental groups. These data indicate that motor performance parameters and kinetic speed remained independent of glycemic status, allowing for an unbiased interpretation of recognition memory indices. Body weight was identified as the main predictor of kinetic performance in the obesity model. In particular, the normoglycemic obese group showed a near-absolute negative correlation between weight and walking speed. The fact that speed is strongly linked to weight, but not to blood glucose levels, suggests that the reductions in exploratory activity observed in the obese groups have a significant mechanical component that should be distinguished from hippocampal-mediated memory deficits (Figure 6I,J).

To our knowledge, this is the first time a correlation analysis has been conducted to discriminate between obesity and diabetes in the ZDF model, focusing on motivational motor function linked to the hippocampus. In this model, the onset of hyperglycemia in young-adult rats did not cause hippocampal damage, possibly because redox balance was maintained. However, this study has limitations that must be addressed in future investigations, including the molecular mechanisms underlying hippocampal damage in obesity (inflammatory status mediated by interleukins, cytokines, and chemokines), neurotransmitters alterations, neuronal morphometry studies, plasticity and eLTP, the role of glia, neurotrophic factor profiles, and the study of other brain regions involved in motivation and motor execution (NAc, amygdale, prefrontal cortex, and cerebellum). Also, this study is cross-sectional; conclusions are limited by the lack of information about the temporal progression of neurodegeneration. Despite the robustness of our findings, certain limitations must be acknowledged when translating them into clinical practice. The ZDF rat is a monogenic model of leptin resistance, whereas human T2D is a complex polygenic condition. Furthermore, while the hippocampal damage and recognition memory deficits reported here align with clinical observations of ‘diabetic encephalopathy,’ the accelerated metabolic timeline of rodents may not fully reflect the decades-long progression of the disease in humans. Future studies should integrate these findings with longitudinal human neuroimaging and metabolic profiling to validate these biomarkers in a clinical setting.

## 5. Conclusions

In summary, ZDF rats at 13 weeks showed early hyperglycemia, similar to T2D in humans, along with obesity. ZDF-HG rats also exhibited severe hepatic insulin resistance and beta-cell dysfunction. ZDF-NG and ZDF-HG presented dyslipidemia. Additionally, both obese groups showed reduced motor activity and short- and long-term recognition memory, with fewer hippocampal neurons, without redox balance decline, although hippocampal ROS was significantly elevated and GR activity was reduced in both obese phenotypes. The correlation analysis demonstrated that obesity did not affect muscle mass or mobility, but it did lead to discouragement behavior. In conclusion, obesity triggers hippocampal neurodegenerative processes that could disrupt the neural circuitry regulating motivation and impulsivity.

## Figures and Tables

**Table 1:** Zoometric parameters of the 3 groups analyzed and statistical analysis. Values correspond to the mean ± SEM (standard error of the mean). A two-way ANOVA with a significance level set at *p* ≤ 0.05 was performed. Significant compared with LEAN group ^$^ *p* < 0.005, ^#^ *p* < 0.0005, ^&^ *p* < 0.0001. ▲ means an increase compared with the control group.

Parameters	LEAN (n = 10)	ZDF-NG (n = 10)	ZDF-HG (n = 10)
Weight (g)	250.4 ± 10.5	▲ 450 ± 50 ^#^	▲ 439.3 ± 17.6 ^&^
Length (cm)	20.3 ± 0.46	21.3 ± 0.25	21.9 ± 0.59
Abd. perimeter (cm)	15.0 ± 0.24	▲ 20.8 ± 1.3 ^&^	▲ 21.3 ± 0.48 ^&^
Peripancreatic fat (g)	0.13 ± 0.01	▲ 2.0 ± 0.08 ^$^	▲ 2.3 ± 0.29 ^&^
Retroperitoneal fat (g)	0.76 ± 0.22	▲ 9.33 ± 0.5 ^$^	▲ 11.67 ± 1.4 ^&^
Epididymal fat (g)	1.56 ± 0.26	▲ 7.68 ± 0.96 ^$^	▲ 9.28 ± 0.92 ^&^
Muscle (g)	1.35 ± 0.19	1.22 ± 0.02	1.49 ± 0.13

**Table 2:** Lipidic parameters of the 3 groups analyzed and statistical analysis. Values correspond to the mean ± SEM (standard error of the mean). A two-way ANOVA with a significance level set at *p* ≤ 0.05 was performed. ▲ means an increase compared with the control group, ▼ means a decrease compared with control group.

Parameters	LEAN (n = 10)	ZDF-NG (n = 10)	ZDF-HG (n = 10)
Total Cholesterol (mg/dL)	113.0 ± 8.8	▲ 205.6 ± 21.6 ^&^	▲ 276.8 ± 25.5 ^&^
VLDL (mg/dL)	14.0 ± 2.3	▲ 66.0 ± 11.0 ^#^	▲ 38.0 ± 13.1 *
HDL (mg/dL)	29.9 ± 1.2	▼ 21.8 ± 4.9 *	▲ 36.6 ± 6.4 *
LDL (mg/dL)	69.1 ± 5.3	117.8 ± 5.5	▲ 202.2 ± 61.0 ^$^
Triglycerides (mg/dL)	54.1 ± 8.4	▲ 262.8 ± 57.2 ^$^	▲ 199.0 ± 56.6 *

Significant respect to LEAN group * *p* < 0.05, ^$^ *p* < 0.005, ^#^ *p* < 0.0005, ^&^ *p* < 0.0001.

## Data Availability

The original contributions presented in this study are included in the article. Further inquiries can be directed to the corresponding authors.
